# Impact of multidisciplinary simulation training on endovascular thrombectomy: Workflow, patient outcomes and anaesthetic management

**DOI:** 10.1177/15910199251336952

**Published:** 2025-06-17

**Authors:** Caroline G Fugelli, Martin W Kurz, Britt S Hansen, Soffien Ajmi, Jan T Kvaløy, Lars Fjetland, Cecilie Grøtteland, Snorre Eikeland, Hege Ersdal

**Affiliations:** 1 Department of Anaesthesia, 60496 Stavanger University Hospital, Stavanger, Norway; 2 Faculty of Health Sciences, 56627 University of Stavanger, Stavanger, Norway; 3 Department of Neurology, 60496 Stavanger University Hospital, Stavanger, Norway; 4 Department of Clinical Medicine, 1658 University of Bergen, Bergen, Norway; 5 Department of Mathematics and Physics, 56627 University of Stavanger, Stavanger, Norway; 6 Stavanger Medical Imaging Laboratory (SMIL), Department of Radiology, 60496 Stavanger University Hospital, Stavanger, Norway; 7 Business Intelligence Unit (Department of Analyses), 60496 Stavanger University Hospital, Stavanger, Norway; 8 Department of Simulation-based Learning, 60496 Stavanger University Hospital, Stavanger, Norway

**Keywords:** Education and training, multidisciplinary simulation training, acute ischaemic stroke, endovascular thrombectomy, nonoperating room anaesthesia

## Abstract

**Background:**

Endovascular thrombectomy (EVT) is a time-sensitive treatment for acute stroke patients. This study was conducted to evaluate the impact of multidisciplinary simulation training on workflow, patient outcomes, and anaesthetic management during EVT.

**Methods:**

This pre-post interventional study treated 244 stroke patients with EVT (55 pre- and 189 postintervention) between May 2016 and November 2021. A multidisciplinary in situ EVT simulation training programme, including a new EVT protocol with a higher blood pressure target range, was implemented in 2017. We assessed the following variables: (1) Workflow metrics: Process times, revascularisation success, and complications; (2) patient outcomes: Symptomatic intracerebral haemorrhage, functional outcomes at 90 days, and the National Institute of Health Stroke Scale postprocedure; and (3) anaesthetic management: Systolic blood pressure (SBP) thresholds, adherence to protocol, and the conversion rate from conscious sedation to general anaesthesia.

**Results:**

The postintervention workflow improved significantly, with a reduction in the median groin puncture-to-reperfusion time from 76 to 53 min (*p* = 0.003) and in the door-to-angio suite arrival time from 54 to 35 min (*p* < 0.001). Other EVT workflow metrics and patient outcomes remained unchanged. Postintervention haemodynamic management significantly changed with increasing median SBP outside protocol thresholds (14 vs. 28.5 min, *p* = 0.003). A variety of different combinations of anaesthetics were used for conscious sedation.

**Conclusions:**

Multidisciplinary simulation training improved EVT workflow times, highlighting its potential to optimise processes. However, the lack of significant improvement in patient outcomes and anaesthetic management suggests the need for a stronger focus on anaesthesia in future training to optimise EVT outcomes.

## Introduction

Stroke, the second leading cause of death worldwide, affects more than 12 million people each year, and its incidence continues to rise.^1^ Alongside this growing burden, stroke treatment options have undergone a revolution. Intravenous thrombolysis (IVT) and endovascular thrombectomy (EVT) for acute ischaemic stroke with large-vessel occlusion have been firmly established as standard care. These treatments must be administered promptly, as the therapeutic benefits are highly time-sensitive.^2,3^ Delays can result in significant penumbra loss and, consequently, poorer patient outcomes. Our previous studies have shown that simulation training for IVT significantly reduces treatment times, ultimately leading to lower stroke mortality rates and improved patient outcomes.^4^ EVT is a more complex procedure that necessitates a high level of expertise from interventional radiologists and seamless teamwork among a diverse team of specialists, including neurologists, radiographers, and anaesthesia professionals. Anaesthesia assistance is crucial for effectively extracting the thrombus, and minimising anaesthesia delay cannot be overstated. Maintaining haemodynamic stability is another priority in anaesthetic management during EVT.^5,6^

Anaesthesia for EVT is categorised as a nonoperating room anaesthesia (NORA) procedure, which poses unique challenges for anaesthesia providers and entails potential risks for patients. These challenges include limited space and access to the patient, constrained anaesthesia resources, high stress levels, a heavy workload, and collaboration in an unfamiliar team.^7–10^ It is suggested that simulation training be implemented to improve the quality and safety of the NORA while addressing the aforementioned obstacles.^9^ As part of a quality improvement (QI) project, we have implemented simulation training for EVT since 2017. The simulation training was designed, planned, and led by a team that did not include anaesthesia professionals.

Considering the critical role of anaesthesia in EVT and the multidisciplinary nature of the procedure, this study aimed to evaluate the impact of our multidisciplinary in situ EVT simulation training programme, which was introduced in 2017, on clinical workflow efficiency, patient outcomes, and anaesthetic management during EVT procedures.

## Methodology

### Study design

The study is a single-centre, retrospective, pre-post intervention analysis that received approval from the Regional Committee for Medical and Health Research (2018/1895). The study is reported against SQUIRE (Standards for Quality Improvement Reporting Excellence Guidelines).^11^

### Research setting

Annually, approximately 450 patients with acute stroke are admitted to our hospital, with approximately 11% receiving EVT since its introduction in 2009. The multidisciplinary EVT team operates on a 24/7 on-call basis and consists of emergency room (ER) nurses, neurologists, radiographers, general interventional radiologists, nurse anaesthetists, and general anaesthesiologists. The protocol involves an immediate response to an alert system, facilitating the swift transfer of patients to the angio suite by a neurologist and ER nurses. Upon arrival, the patient is received by awaiting anaesthesia and radiology professionals. Conscious sedation was the recommended method of anaesthesia for EVT, with general anaesthesia reserved for eligible patients (Supplemental Table S1).

### Quality improvement project

In February 2017, our QI team, consisting of a neurology registrar, senior consultant stroke physician, and consultant radiologist, planned and implemented a QI project to improve acute stroke treatment at our hospital.^4^ This project began with an overhaul of the in-hospital stroke treatment protocol and the introduction of in situ simulation training for IVT treatment. Subsequently, in November 2017, we refined the EVT protocol and launched EVT simulation training, focusing on enhancing protocol knowledge, compliance, task distribution, and team efficiency. Notably, the anaesthesia protocol was updated to reflect evidence-based systolic blood pressure (SBP) thresholds, adjusting from 110–160 mmHg to a more permissive range of 140–180 mmHg.^12^ During the following study period, weekly in-hospital acute stroke treatment training sessions, including both IVT and EVT, were held once or twice annually in clusters of four months. All EVT team members participated in the simulation training; however, only the QI team planned, operated, and facilitated the sessions. In EVT simulations, a manikin combined with a virtual simulator was used as a simulated patient (SimMan Vascular, Laerdal Medical, Stavanger, Norway, and Mentice VIST^®^ G5 simulator, Mentice AB, Gothenburg, Sweden). Continuous evaluation and periodic revision of the stroke treatment protocol were performed as needed; that is, the threshold for SpO_2_ was changed from 96% to 93% in November 2018, according to new guidelines.^13^ Moreover, during a focused 5-month period from 2019 to 2020, our general interventional radiologists engaged in dedicated technical practice sessions using a virtual simulator.^14^ The details of the QI project and corresponding revisions can be found in the Supplemental Table S2.

### Data collection and variables

This study included patients with acute ischaemic stroke who underwent EVT at our hospital. Data on patient demographics, stroke onset as defined by the National Stroke Registry, treatment modalities, and outcomes were extracted from the hospital's local and national stroke registries.^15^ Intraprocedural data such as time metrics, patient vital parameters, and anaesthetics were automatically extracted from hospital anaesthesia electronic medical records (AEMRs) (Picis Anaesthesia Manager, Picis Clinical Solutions, Inc., Wakefield, Boston, MA, USA) by the hospital's analysis department. Any instances of extreme outliers or notable remarks within the AEMR prompted a manual review of relevant records. The aim of this analysis was to compare the collected data across two distinct periods before and after the initiation of the intervention in November 2017. Given that a specific anaesthesia protocol for EVT was not established until May 2016, the preintervention data collected commenced from that date onwards.

The EVT workflow was assessed using various time metrics, the rate of successful revascularisation (TICI score 2b-3), and the frequency of intraprocedural complications. For patient outcomes, our metrics included the incidence of symptomatic intracerebral haemorrhage within the first 24 h following the procedure, achievement of a good functional outcome signifying functional independence 90 days after stroke onset (modified Rankin scale (mRS) 0–2), occurrence of the worst functional outcome, either being bedridden or deceased, 90 days poststroke (mRS 5–6), and National Institute of Health Stroke Scale (NIHSS) scores both postprocedure and at the time of discharge.

Anaesthetic management was assessed based on critical criteria, including haemodynamic stability, adherence to the recommended anaesthesia protocol, and the rate at which primary conscious sedation needed to be converted to general anaesthesia. Haemodynamic stability was evaluated by monitoring the absolute and relative duration during which the patient's SBP remained outside the thresholds specified in the hospital's EVT protocol. Although invasive arterial pressure monitoring is the norm for EVT procedures, noninvasive blood pressure measurements were employed in cases in which invasive monitoring was not feasible (Supplemental Table S3).

Patients with incomplete data on total procedural time or haemodynamic measurement in AEMR, as well as those who underwent long and complicated procedures (>240 min), were excluded from the anaesthetic management analysis. For the workflow process and patient outcomes analyses, we excluded patients with a pre-EVT modified Rankin Scale (mRS) score ≥2, those who underwent multiple EVT procedures during the same hospital stay, died of cancer within 3 months, or had procedures exceeding 240 min. The 240-min cutoff was chosen to avoid extreme outliers.

### Statistical analysis

Sample size calculations were performed based on SBP. A clinically significant difference was defined as a 13 percentage point absolute decrease in the proportion of time with an SBP outside the defined thresholds during procedural anaesthesia care. Although there were no directly comparable studies for the SBP variable, we deemed this improvement achievable based on findings from specialised neuroanaesthesia centres and practical considerations.^16^ Based on an observed standard deviation of 26 percentage points in the preintervention group, the planned sample size was at least 85 patients in the postintervention group according to a two-sided t-test with a power of 0.8, setting the statistical significance at *p* < 0.05.

Categorical variables are presented as absolute numbers and frequencies. Continuous data were non-Gaussian and are presented as medians and first and third quartiles. Differences between the groups were checked using the Mann‒Whitney U test for continuous variables and the Pearson chi-square test for categorical data. In addition, to illustrate changes in the time from groin puncture to successful reperfusion as a continuous process over time, we used run charts.^17^ A multivariable logistic regression analysis was used for the outcome of mRS 0–2 in the postintervention group. Variables were included in the model based on clinical relevance. Statistical significance was defined for hypothesis tests as a 2-tailed *p* < 0,05. Analyses were performed using SPSS Statistics version 26 (IBM Cooperation, Armonk, NY, USA) and R version 4.3.3.^18^

## Results

**Figure 1.**
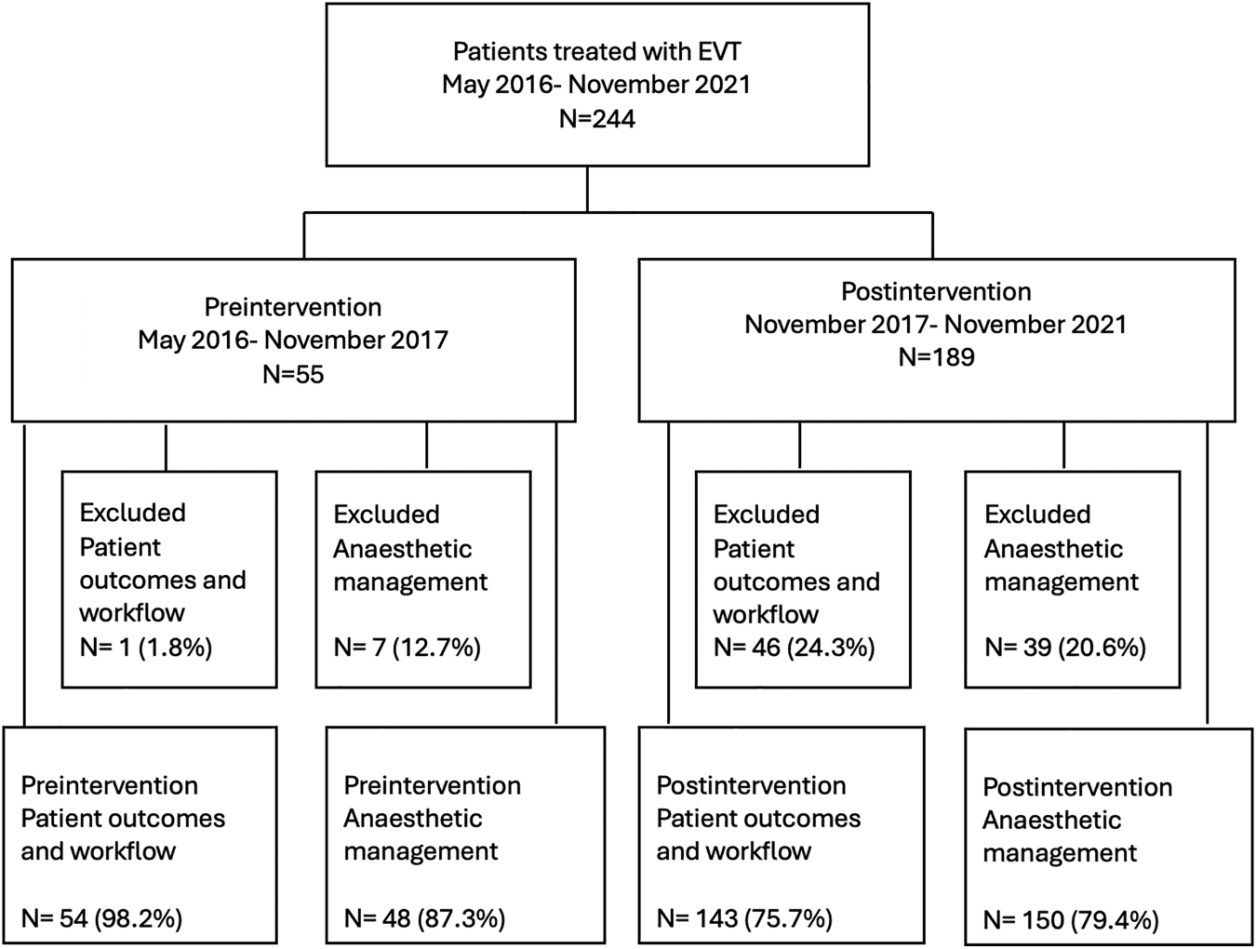
Flow diagram of patients included in the analyses. EVT: endovascular thrombectomy.

Between May 14, 2016, and November 3, 2021, a total of 244 patients were enrolled in the study. Of these, 55 underwent EVT treatment during the preintervention period and 189 during the postintervention period (Figure 1). Forty-six patients were excluded from the anaesthetic management analysis (7 patients preintervention and 39 patients postintervention), and 47 patients from the analyses of workflow process and patient outcomes (1 patient preintervention and 46 patients postintervention).

There were no statistically significant differences in patient characteristics when comparing the pre- and post-intervention groups with regard to age, ischaemic stroke risk factors, NIHSS score at admission, prestroke mRS score, initial Alberta Stroke Programme Early CT score or proportion of patients who were pretreated with IVT prior to EVT (Table 1). The location of the occlusion was also similar across the groups. There were significantly more patients monitored with invasive arterial blood pressure monitoring in the postintervention period (*p *< 0.001).

**Table 1.** Comparison of patient characteristics before and after the intervention.

Workflow process and patient outcomes cohort	Preintervention (n = 54)	Postintervention (n=143)	*P* Value
Age (years)	77 (62.8-84.0)	74 (62.0-81.0)	0.30
Female	29 (53.7)	61 (42.7)	0.17
Comorbid conditions			
Atrial fibrillation	22 (40.7)	61 (42.7)	0.81
Diabetes mellitus	7 (13.0)	22 (15.4)	0.67
Hypertension	36 (66.7)	79 (55.2)	0.15
Previous myocardial infarction	14 (25.9)	24 (16.8)	0.15
Previous cerebral infarction	10 (18.5)	17 (11.9)	0.23
Clinical variables			
Initial NIHSS	18 (11-23.3)	16 (11-21)	0.33
Initial mRS^†^	0.0 (0.0-0.0)	0.0 (0.0-0.0)	0.17
Initial ASPECT score^‡^	9 (7-10)	8 (6-10)	0.20
Bridging thrombolysis	35 (64.8)	94 (65.7)	0.90
Anterior circulation occlusion site	49 (90.7)	132 (92.3)	0.72
Invasive arterial blood pressure monitoring^§^	43 (81.1)	137 (97.9)	<0.001

Anaesthetic management cohort	Preintervention (n = 48)	Postintervention (n = 150)	*P* value
Age (years)	78.5 (69.3–84.8)	74.5 (63.8–82.3)	0.12
Female	27 (56.3)	64 (42.7)	0.10
Comorbid conditions			
Atrial fibrillation	21 (43.8)	68 (45.6)	0.82
Diabetes mellitus	7 (14.6)	28 (18.8)	0.51
Hypertension	32 (66.7)	82 (55.0)	0.16
Previous myocardial infarction	13 (27.1)	24 (16.1)	0.09
Previous cerebral infarction	9 (18.8)	18 (12.1)	0.24
Clinical variables			
Initial NIHSS	16.5 (11.0–21.8)	16.0 (10.5–21.5)	0.84
Initial mRS^¶^	0.0 (0.0–0.0)	0.0 (0.0–0.0)	0.41
Initial ASPECT score Anterior circulation occlusion site	9.0 (7.0–10.0) 44 (91.7)	8.0 (6.0–10.0) 134 (89.3)	0.14 0.64
Bridging thrombolysis	33 (68.8)	90 (60.0)	0.28
Invasive arterial blood pressure monitoring	39 (81.3)	148 (98.7)	< 0.001

Abbreviations: ASPECT: Alberta stroke program early CT score; mRS: modified Rankin scale; NIHSS: national institutes of health stroke scale/score.

Notes: Numbers are reported as numbers (percentages) for categorical variables and medians (quartiles) for continuous variables.

† N = 52 preintervention, N = 141 postintervention.

‡ N = 54 preintervention, N = 141 postintervention.

§ N = 53 preintervention, N = 140 postintervention.

¶ N = 47 preintervention, N = 145 postintervention.

**Figure 2.**
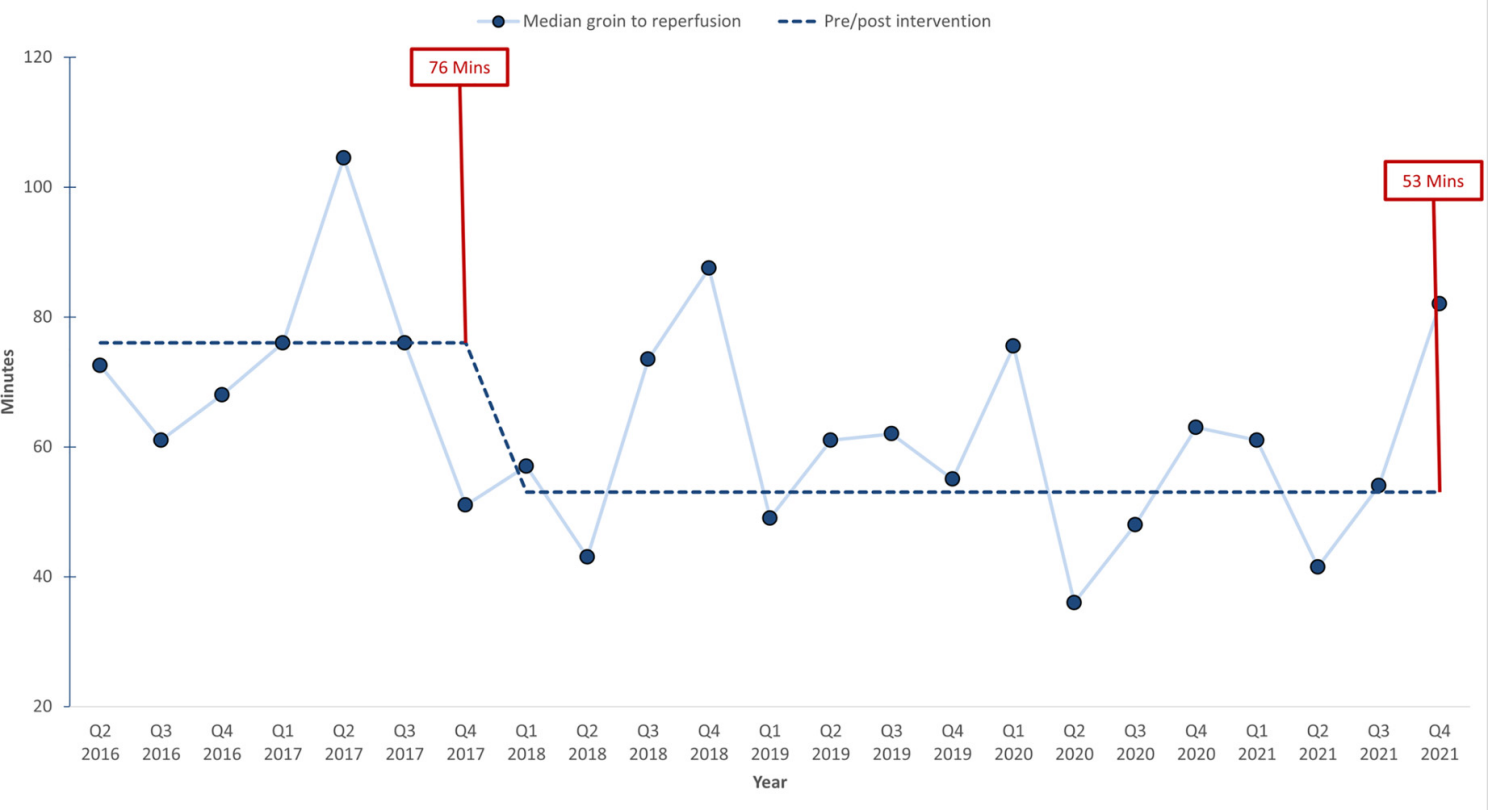
Run chart illustrating groin puncture-to-reperfusion time.

In our analysis of workflow efficiency, we observed notable reductions in critical time intervals, reflecting significant improvements in patient processing times. In the pre-EVT phase, which did not involve anaesthesia professionals, the time from hospital arrival to angio suite entry significantly decreased from 54 min to 35 min. During the EVT procedure, which involved anaesthesia professionals, the duration from groin puncture to successful reperfusion decreased from 76 to 53 min (Table 2). This change is illustrated in Figure 2. In contrast, certain intervals within the EVT process, such as the time from angio suite entry to groin puncture, and intervals outside the EVT process, such as the time from the onset of symptoms to arrival at the angio suite, remained unchanged before and after the intervention (Table 2). The percentage of technically successful reperfusions changed from 77.8% to 86.7%, but this change was not statistically significant (*p* = 0.12). Similarly, the rates of interventional complications and the incidences of ICH remained consistent throughout the study period, with no significant changes observed.

**Table 2.** Comparison of the EVT workflow and patient outcomes before and after the intervention.

Variables	Preintervention (n = 54)	Postintervention (n = 143)	*P* value
EVT workflow			
Onset-Angio suite arrival (min)^†^	119.5 (85.3-245.0)	119.5 (79.3-207.3)	0.86
Onset-reperfusion (min)^‡^	220 (182-272.8)	210 (163.0-295.3)	0.73
Door-Angio suite arrival (min)^§^	54 (35-99)	35 (25.5-50)	<0.001
Angio suite arrival-groin puncture (min)	15 (9-21)	15 (10-21.8)	0.75
Groin puncture-reperfusion^¶^	76 (51.5-99.5)	53 (37-79)	0.003
TICI score 2b-3	42 (77.8)	124 (86.7)	0.12
Interventional complications^††^	7 (14)	27 (19.6)	0.38
Patient outcomes			
ICH	5 (9.3)	5 (3.5)	0.10
mRS 0-2^‡‡^	20 (38.5)	70 (49.6)	0.17
mRS 5-6^‡‡^	13 (25)	19 (13.5)	0.06
Initial NIHSS	18 (11-22.3)	16 (11-21)	0.33
NIHSS postintervention	15 (4.8-23.3)	9 (3-17)	0.01
NIHSS difference at 24 h	2 (-5.3-7.3)	4 (0-11.3)	0.02
NIHSS at discharge^§§^	3 (0-15)	6 (1-11)	0.89

Abbreviations: EVT: endovascular thrombectomy; ICH: intracerebral haemorrhage; mRS: modified Rankin scale; NIHSS: national institutes of health stroke scale/score; TICI: thrombolysis in cerebral infarction.

Notes: Numbers are reported as numbers (percentages) for categorical variables and medians (quartiles) for continuous variables.

Numbers are reported as numbers (percentages) for categorical variables and medians (quartiles) for continuous variables. Time variables are reported as minutes.

† N = 36 preintervention, N = 113 postintervention.

‡ N = 36 preintervention, N = 102 postintervention.

§ N = 43 preintervention, N = 125 postintervention.

¶ N = 45 preintervention, N = 127 postintervention.

†† N = 50 preintervention, N = 138 postintervention, excluding patients with pre mRS > 2.

‡‡ N = 52 preintervention, N = 141 postintervention.

§§ N = 43 preintervention, N = 131 postintervention.

Neurological outcomes improved significantly 24 hours post-EVT, with median NIHSS scores decreasing from 15 in the preintervention group to 9 in the postintervention group (*p *= 0.01). However, this improvement was not reflected as a statistically significant difference at discharge (median NIHSS 3 preintervention vs. 6 postintervention, *p *= 0.90) or measured by mRS (Table 2). The proportion of patients with good clinical outcomes (mRS: 0–2) changed from 38.5% preintervention to 49.6% postintervention (*p *= 0.17), and the proportion of patients withworst clinical outcomes (mRS: 5–6) changed from 25% preintervention to 13.5% postintervention (*p *= 0.06).

Notably, 14 different combinations of anaesthetics were utilised for conscious sedation before the simulation training was introduced, whereas 13 combinations were utilised afterwards, indicating no significant change in the variety of anaesthetics employed. The rate of adherence to the recommended anaesthetics for conscious sedation and general anaesthesia did not significantly improve in the postintervention group (Table 3). The percentage of patients who converted from conscious sedation to general anaesthesia decreased nonsignificantly from 16.7% before training to 12% after training. The ability to maintain SBP within the recommended threshold decreased in the postintervention period, utilising updated SBP thresholds of 140–180 mmHg from the prior 110–160 mmHg. The median time of SBP outside the thresholds increased by 15 min compared to the preintervention period, constituting a 20% increase in the proportion of time with SBP outside the thresholds during procedural anaesthesia care. Notably, every 10 min spent outside the SBP thresholds in the postintervention group was associated with a 17% reduction in the odds of achieving a favourable neurological outcome (OR 0.83 (CI = 0.71–0.97), *p* = 0.02). This association remained significant after adjusting for stroke onset, stroke severity, age and comorbidities (OR 0.80 (CI = 0.65–0.98), *p* = 0.03). However, when adjusting for successful reperfusion, the association was no longer significant (OR 0.83 (CI = 0.68–1.03), *p* = 0.09).

**Table 3.** Comparison of anaesthetic management before and after the intervention.

Variables	Preintervention (n = 48)	Postintervention (n = 150)	*P* value
Primary sedation	42 (87.5)	115 (76.7)	0.11
Compliance with recommended anaesthetics, sedation^†^	18 (43.9)	49 (43.4)	0.95
Compliance with recommended anaesthetics, GA^‡^	3 (21.4)	14 (27.5)	0.65
Conversion from sedation to GA	8 (16.7)	18 (12)	0.41
Procedural time within anaesthesia care (min)	92.5 (67.3–117.8)	82.5 (57.8–111)	0.23
SBP outside threshold (min)	14 (7.3–35.8)	28.5 (14.8–43)	0.003
SBP outside threshold, n (%)	17.2 (8–37.7)	37 (20–54.7)	< 0.001
Hypotension, n (%)	2.5 (0–12.7)	14.6 (5.8–41.2)	< 0.001
Hypertension, n (%)	5.5 (0–21.6)	4.6 (0.3–20.3)	0.60

Abbreviations: GA: general anaesthesia; SBP: systolic blood pressure.

Notes: Numbers are reported as numbers (percentages) for categorical variables and medians (quartiles) for continuous variables.

† N = 41 preintervention, N = 113 postintervention.

‡ N = 14 preintervention, N = 51 postintervention.

## Discussion

EVT simulation training significantly reduced the clinical time from groin puncture to reperfusion, highlighting the effectiveness of simulation training in improving procedural efficiency. However, functional outcomes did not improve significantly. While good outcomes (mRS 0–2) were more frequent postintervention, and poor outcomes (mRS 5–6) declined, these difference were not statistically significant. The observed variety in anaesthesia combinations used and the significant duration of SBP readings outside the defined thresholds postintervention suggest that these factors may contribute to a lack of improvement in patient outcomes and are potential targets for improvement in simulation training.

Several studies have demonstrated that simulation training can improve clinical workflows for acute stroke treatment. Most of this research has focused on IVT or EVT until groin puncture. However, while improvements in process times are commonly observed, only one study, the one by Ajmi et al.,^4^ has shown a direct clinical benefit of simulation training on patient outcomes. The majority of studies, including the STREAM trial, showed enhanced procedural efficiency but did not report patient outcomes.^19^

To our knowledge, this is the first study to report an improvement in the intervention time from groin puncture to reperfusion in the context of EVT through simulation training. Although our simulation training did not affect the total time from symptom onset to reperfusion, the duration from groin puncture to successful reperfusion significantly decreased from 76 to 54 min. While the total time from symptom onset to recanalisation is the most important time interval with regard to patient outcomes, the interval between groin puncture and reperfusion is strongly associated with favourable outcomes.^20–22^ This interval serves as a surrogate marker of the effects of EVT simulation training because it reflects the technical ability of the interventional radiologist and the technical aspects of EVT, including team interplay and anaesthesia performance.^23^ Despite these comprehensive improvements in procedural time metrics and the substantial increase in successful reperfusion rates, we did not observe improvements in clinical outcomes; this may be because the total time from symptom onset to reperfusion did not change. Additionally, this factor may highlight the influence of factors other than procedural time metrics on EVT outcomes. Haemodynamic stability during EVT may be one of these factors. A post hoc analysis of the ASTER trial, which compared contact aspiration versus stent retriever for successful recanalisation, showed that greater variability in systolic BP, diastolic BP, and mean arterial pressure was negatively associated with favourable outcomes, irrespective of the patient's collateral status. Additionally, hypotension was negatively associated with outcomes in patients with poor collateral status.^24^

Maintaining haemodynamic stability during EVT is a crucial aspect of anaesthetic management, and haemodynamic instability is one of the most common complications in NORA procedures, highlighting it as a challenge in EVT.^10^ EVT stands out within acute stroke treatment due to its reliance on close collaboration between interventional radiologists and anaesthesia professionals in the coordinated performance of practical tasks and skills to achieve successful outcomes.^25,26^ The simulation training of interventional radiologists can improve their technical performance.^14^ Such improvements can result in improved time intervals, as observed in our study, or improved reperfusion rates and fewer intraprocedural complications, which we observed as nonsignificant changes. However, the management of anaesthesia during EVT has only been evaluated in clinical studies unrelated to simulation training. A significant focus has been on the influence of haemodynamic control, where hypotension is particularly undesirable and reduces the chance of good functional outcome.^16,27^ Protocol revision limited the comparison of haemodynamic stability between the groups in our study, but the lack of significant improvements suggested an inadequate emphasis on haemodynamic parameters in our simulation training. This inadequacy affects the ability to deliver care according to existing procedures. The low compliance with the anaesthesia protocol for EVT and the high conversion rate from sedation to general anaesthesia compared with other studies further support the interpretation of the inadequate focus on anaesthetic management in our EVT simulation training.^28,29^

Our simulation training was designed and led by nonanaesthesia professionals, which likely explains the lack of integration of challenges relevant to anaesthetic management into simulation training. Teamwork in NORA procedures, such as EVT, is known to be stressful for anaesthesia professionals due to the lack of understanding between anaesthesia and nonanaesthesia professionals. Anaesthesia professionals have reported this as a threat to patient safety and workflow in NORA locations, such as the angio suite.^7,9,30^ Our findings highlight the importance of involving anaesthesia professionals in the design of simulation training for EVT to provide relevant input that could improve anaesthetic management. The association between maintaining SBP within protocol recommendations and favourable functional outcomes in the postintervention group suggests that focusing on anaesthesia in simulation training has the potential to improve patient outcomes.

Our study is limited by its single-centre, retrospective, observational design and the unbalanced number of patients pre- and postintervention, which may have been too small to detect differences in patient outcomes. Additionally, approximately 19% of the initial patient population was excluded from the analyses. This exclusion may introduce selection bias. The protocol revision also involved several factors relevant to anaesthetic management, necessitating caution when drawing conclusions about its quality. Despite these limitations, this is the first study to assess the effects of simulation training on clinical EVT teamwork. Given that most hospitals require anaesthesia for EVT, our findings are relevant for planning and designing simulation training in other EVT centres. Including anaesthesia professionals in the design and execution of EVT simulation training is essential, as they provide critical knowledge for delivering safe and efficient anaesthesia, which can impact patient outcomes. Further research is needed to study the impact of increased emphasis on anaesthetic management within EVT simulation training on patient outcomes post-EVT.

This study demonstrated that EVT simulation training significantly improved procedural time metrics but did not necessarily translate to better overall patient outcomes. This can be due, in part, to the lack of observed improvement in anaesthetic management during the study. These findings indicate the need to incorporate anaesthesia considerations into future EVT simulation training programmes to provide more comprehensive stroke care.

## Supplemental Material

sj-docx-1-ine-10.1177_15910199251336952 - Supplemental material for Impact of multidisciplinary simulation training on endovascular thrombectomy: Workflow, patient outcomes and anaesthetic management Supplemental material, sj-docx-1-ine-10.1177_15910199251336952 for Impact of multidisciplinary simulation training on endovascular thrombectomy: Workflow, patient outcomes and anaesthetic management by Caroline G Fugelli, Martin W Kurz, Britt S Hansen, Soffien Ajmi, Jan T Kvaløy, Lars Fjetland, Cecilie Grøtteland, Snorre Eikeland and Hege Ersdal in Interventional Neuroradiology
