# Supplementary material for: Impact of multidisciplinary simulation training on endovascular thrombectomy: Workflow, patient outcomes and anaesthetic management
Source: Interv Neuroradiol. 2025 Jun 17:15910199251336952. Online ahead of print. doi: 10.1177/15910199251336952 (PMC12174582; doi:10.1177/15910199251336952)
Supplement: sj-docx-1-ine-10.1177_15910199251336952 - Supplemental material for Impact of multidisciplinary simulation training on endovascular thrombectomy: Workflow, patient outcomes and anaesthetic management [file sj-docx-1-ine-10.1177_15910199251336952.docx]

**Supplemental material**

**Table S1.** Anaesthesia methods and anaesthetics used in the EVT protocol

|  | Preintervention | Postintervention |
| --- | --- | --- |
| Conscious sedation as first choice | | |
|  | Fentanyl 25-50 mikrogr. and midazolam 0,5 mg. bolus iv | Propofol infusion 1-2 mg/kg/h and fentanyl 25-50 mikrog. bolus iv |
| General anaesthesia as second choice. Indicated when;   - Patients are unconscious or threatened airway - Patients have nausea and/or vomiting - Not cooperating with restless behaviour - Posterior occlusion | | |
|  | Sevoflurane inhalation and remifentanil infusion 0,05-0,1 mikrog/kg/min iv. | Propofol infusion 1-3 mg/kg/h and remifentanil infusion 0,05-0,1 mikrog/kg/min iv. |

Abbreviations: EVT: endovascular thrombectomy

**Table S2.** Quality improvement project revisions

| Process intravenous thrombolysis | |
| --- | --- |
| 2017-feb | Prenotification of the in-hospital stroke treatment team through a dedicated ‘stroke thrombolysis’ alarm |
|  | Direct transport from prehospital ambulance to CT lab |
|  | Patient preparation during in-hospital transport |
|  | Whenever thrombolysis treatment is decided, blood samples should be collected after intravenous thrombolysis administration |
|  | Administration of intravenous thrombolysis bolus dose in the CT lab. |
| In situ simulation training intravenous thrombolysis treatment | |
| 2017-feb | Implementing live patient-based simulation for the in-hospital stroke team responsible for intravenous thrombolysis |
| Process EVT | |
| 2017-nov | Prenotification of the in-hospital EVT team through a dedicated EVT alarm |
|  | Defining the neurologist as team leader in angio suite |
|  | Developing and implementing “Safe Thrombectomy” checklist |
|  | Radiologist responsible for inserting an invasive arterial line into the patient's femoral artery prior to groin puncture |
|  | Patient urinary catheter insertion in the angio suite is the responsibility of an emergency room nurse |
| In situ simulation training EVT | |
| 2017-nov | Implementing manikin-based simulation with SimMan Vascular and Mentice VIST® G5 simulator (Mentice AB, Gothenburg, Sweden) in angio suite for the multidisciplinary EVT team |
| 2019- oct/nov | Implementing e-learning program for all IVT and EVT team members |
| Anaesthesia | |
| 2017-nov | Developing and implementing use of “action cards” for anaesthesia team members |
|  | Protocol change of anaesthetics (see Table 1) |
|  | Protocol change from SBP 110-160 (160-185 was allowed for a few minutes) mmHg to 140-180 mmHg for haemodynamic thresholds |
| 2018-nov | Protocol change of respiratory thresholds from SpO2 >=96% to SpO2 >= 93% |

Abbreviations: QI: EVT: endovascular thrombectomy; IVT: intravenous thrombolysis; SBP: systolic blood pressure

**Table S3.** Data collection and definition of selected variables

|  | **Collection in AEMR** | **Definition in database** | **Excluded from measurement** | **Period of measurement** |
| --- | --- | --- | --- | --- |
| Data | | | | |
| Noninvasive blood pressure monitoring | Measured every 3. minute | Defined as outside threshold until a value within threshold is registered | One single measure of extreme value | Collected from the first registration of patients’ vital parameters in the angio suite to the end of the procedure (groin extraction) |
| Invasive blood pressure monitoring | Measured every minute | Defined as outside threshold until a value within threshold is registered | One single measure of extreme value | Collected from the first registration of patients’ vital parameters in the angio suite to the end of the procedure (groin extraction) |
| Variables | | | | |
| Procedural time within anaesthesia care | - First registration of patients’ vital parameters in the angio suite - End of the procedure (groin extraction) | From the first registration of patients’ vital parameters in the angio suite to the end of the procedure (groin extraction) |  |  |
| Haemodynamic variables | Absolute time collected from the first registration of patients’ vital parameters in the angio suite to the end of the procedure (groin extraction) | Relative time reflects the absolute time as a function of procedural time within anaesthesia care |  |  |

Abbreviations: AEMR: anaesthesia electronic medical record
